# PacBio Sequencing and Its Applications

**DOI:** 10.1016/j.gpb.2015.08.002

**Published:** 2015-11-02

**Authors:** Anthony Rhoads, Kin Fai Au

**Affiliations:** 1 Department of Biostatistics, University of Iowa, Iowa City, IA 52242, USA; 2 Department of Internal Medicine, University of Iowa, Iowa City, IA 52242, USA

**Keywords:** Third-generation sequencing, *De novo* assembly, Gene isoform detection, Methylation, Hybrid sequencing

## Abstract

Single-molecule, real-time sequencing developed by Pacific BioSciences offers longer read lengths than the second-generation sequencing (SGS) technologies, making it well-suited for unsolved problems in genome, transcriptome, and epigenetics research. The highly-contiguous ***de novo* assemblies** using PacBio sequencing can close gaps in current reference assemblies and characterize structural variation (SV) in personal genomes. With longer reads, we can sequence through extended repetitive regions and detect mutations, many of which are associated with diseases. Moreover, PacBio transcriptome sequencing is advantageous for the identification of gene isoforms and facilitates reliable discoveries of novel genes and novel isoforms of annotated genes, due to its ability to sequence full-length transcripts or fragments with significant lengths. Additionally, PacBio’s sequencing technique provides information that is useful for the direct detection of base modifications, such as **methylation**. In addition to using PacBio sequencing alone, many **hybrid sequencing** strategies have been developed to make use of more accurate short reads in conjunction with PacBio long reads. In general, **hybrid sequencing** strategies are more affordable and scalable especially for small-size laboratories than using PacBio Sequencing alone. The advent of PacBio sequencing has made available much information that could not be obtained via SGS alone.

## Introduction

While the second-generation sequencing (SGS) technologies have offered vast improvements over Sanger sequencing, their limitations, especially their short read lengths, make them poorly suited for some particular biological problems, including assembly and determination of complex genomic regions, gene isoform detection, and methylation detection. Single-molecule real-time (SMRT) sequencing, developed by Pacific BioSciences (PacBio), offers an alternative approach to overcome many of these limitations. To be concise, it is referred to as “PacBio sequencing” hereinafter, though “SMRT sequencing” is also used by the community.

Unlike SGS, PacBio sequencing is a method for real-time sequencing and does not require a pause between read steps [1]. These features distinguish PacBio sequencing from SGS, so it is classified as the third-generation sequencing (TGS). Here we will summarize the mechanisms and performance of PacBio sequencing. PacBio sequencing offers much longer read lengths and faster runs than SGS methods but is hindered by a lower throughput, higher error rate, and higher cost per base. Since the advantages of PacBio sequencing and SGS are complementary, we will examine hybrid-sequencing strategies that make use of both technologies to overcome the disadvantages of each alone. We will also discuss the applications of PacBio sequencing to various areas of research, including genome, transcriptome, and epigenetics. While reasonable applications of PacBio sequencing to genomics research were initially limited to the finishing of relatively small microbial genomes, PacBio sequencing can now be utilized to study much larger genomes including that of human, thanks to the new computational techniques and improvements in the sequencing technology. The long read lengths of PacBio sequencing also make the technology adept at identification and quantification of isoforms, including novel isoforms, particularly when used in conjunction with SGS. In addition, by monitoring the time between base incorporations, PacBio sequencing kinetic allows for the direct detection of base modifications, such as *N*^6^-methyladenine (m^6^A) and *N*^4^-methylcytosine (m^4^C).

## Mechanism and performance

**Figure 1:**
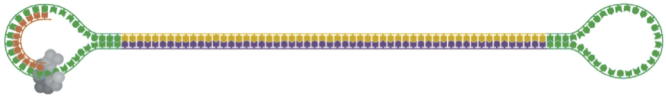
**SMRTbell template** Hairpin adaptors (green) are ligated to the end of a double-stranded DNA molecule (yellow and purple), forming a closed circle. The polymerase (gray) is anchored to the bottom of a ZMW and incorporates bases into the read strand (orange). The image is adapted from [2] with permission from the Oxford University Press.

**Figure 2:**
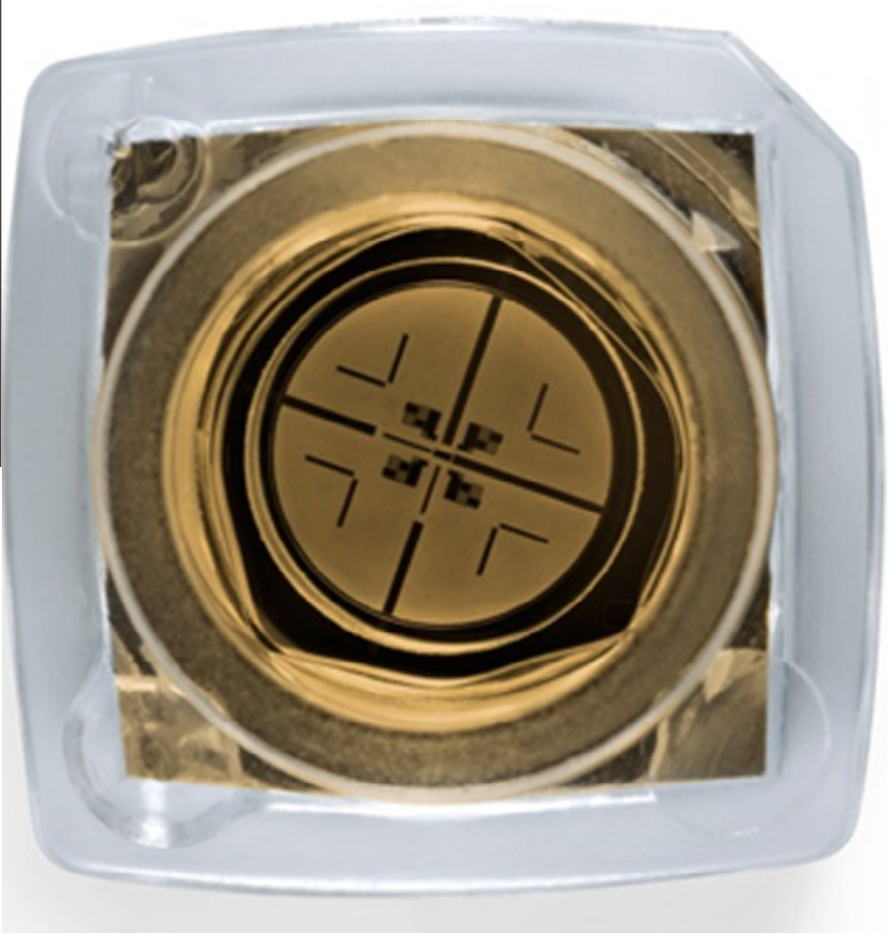
**A single SMRT cell** Each SMRT cell contains 150,000 ZMWs. Approximately 35,000–75,000 of these wells produce a read in a run lasting 0.5–4 h, resulting in 0.5–1 Gb of sequence. The image is adapted with permission from Pacific Biosciences [3]. ZMW, zero-mode waveguide.

**Figure 3:**
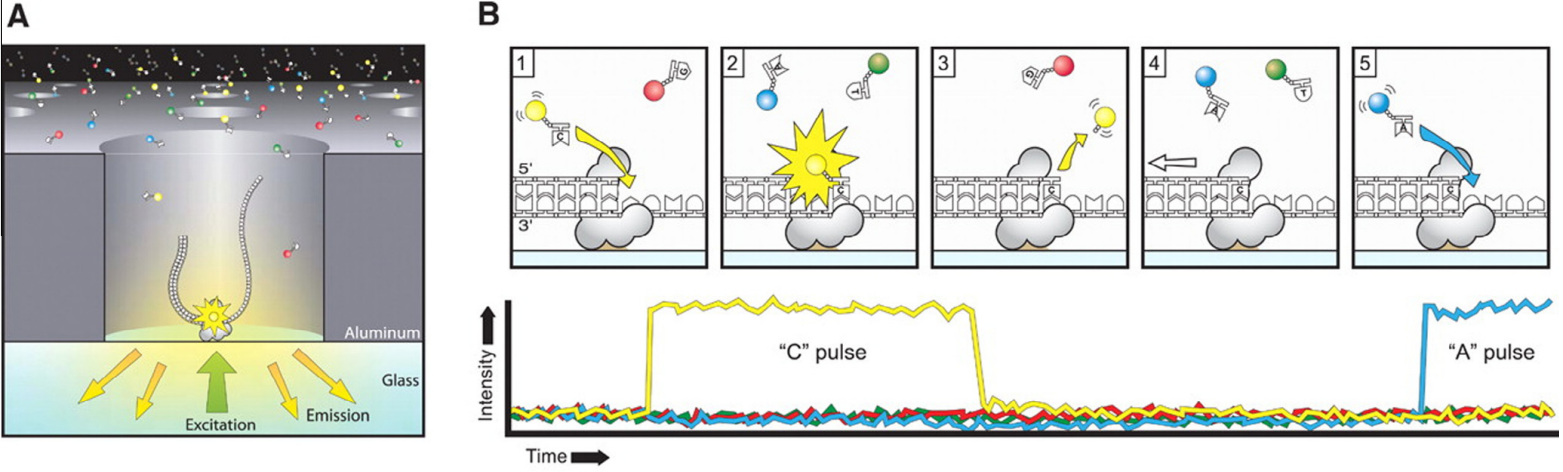
**Sequencing via light pulses** **A.** A SMRTbell (gray) diffuses into a ZMW, and the adaptor binds to a polymerase immobilized at the bottom. **B.** Each of the four nucleotides is labeled with a different fluorescent dye (indicated in red, yellow, green, and blue, respectively for G, C, T, and A) so that they have distinct emission spectrums. As a nucleotide is held in the detection volume by the polymerase, a light pulse is produced that identifies the base. (1) A fluorescently-labeled nucleotide associates with the template in the active site of the polymerase. (2) The fluorescence output of the color corresponding to the incorporated base (yellow for base C as an example here) is elevated. (3) The dye-linker-pyrophosphate product is cleaved from the nucleotide and diffuses out of the ZMW, ending the fluorescence pulse. (4) The polymerase translocates to the next position. (5) The next nucleotide associates with the template in the active site of the polymerase, initiating the next fluorescence pulse, which corresponds to base A here. The figure is adapted from [4] with permission from The American Association for the Advancement of Science.

PacBio sequencing captures sequence information during the replication process of the target DNA molecule. The template, called a SMRTbell, is a closed, single-stranded circular DNA that is created by ligating hairpin adaptors to both ends of a target double-stranded DNA (dsDNA) molecule (Figure 1) [2]. When a sample of SMRTbell is loaded to a chip called a SMRT cell (Figure 2) [3], a SMRTbell diffuses into a sequencing unit called a zero-mode waveguide (ZMW), which provides the smallest available volume for light detection. In each ZMW, a single polymerase is immobilized at the bottom, which can bind to either hairpin adaptor of the SMRTbell and start the replication (Figure 3**A**) [4]. Four fluorescent-labeled nucleotides, which generate distinct emission spectrums, are added to the SMRT cell. As a base is held by the polymerase, a light pulse is produced that identifies the base (Figure3**B**) [4]. The replication processes in all ZMWs of a SMRT cell are recorded by a “movie” of light pulses, and the pulses corresponding to each ZMW can be interpreted to be a sequence of bases (called a continuous long read, CLR). The latest platform, PacBio RS II, typically produces sequencing movies 0.5–4 h in length [5]. Because the SMRTbell forms a closed circle, after the polymerase replicates one strand of the target dsDNA, it can continue incorporating bases of the adapter and then the other strand. If the lifetime of the polymerase is long enough, both strands can be sequenced multiple times (called “passes”) in a single CLR. In this scenario, the CLR can be split to multiple reads (called subreads) by recognizing and cutting out the adaptor sequences. The consensus sequence of multiple subreads in a single ZMW yields a circular consensus sequence (CCS) read with higher accuracy. If a target DNA is too long to be sequenced multiple times in a CLR, a CCS read cannot be generated, and only a single subread is output instead. Because PacBio sequencing takes place in real time, kinetic variation interpreted from the light-pulse movie can be analyzed to detect base modifications, such as methylation [6].

**Figure 4:**
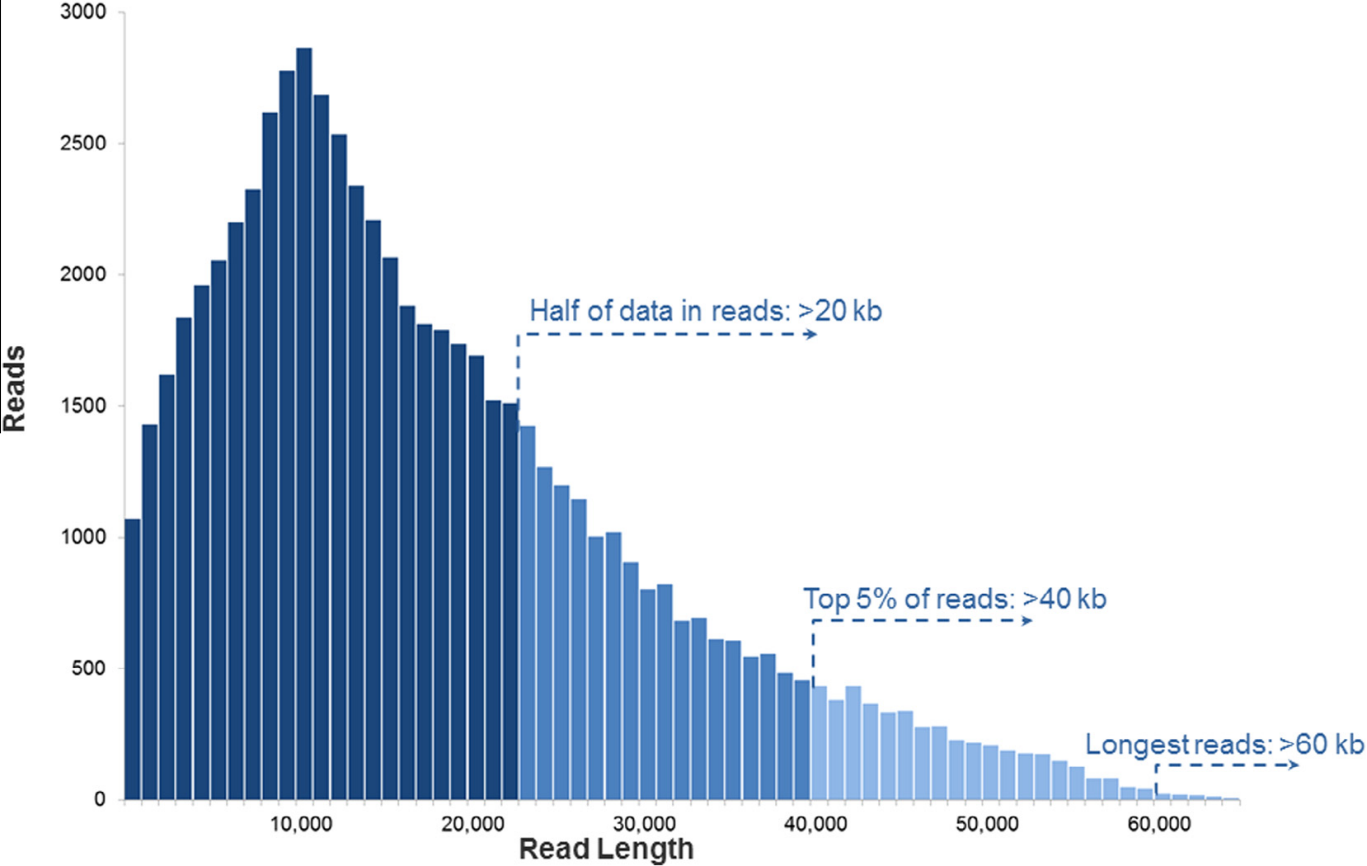
**PacBio RS II read length distribution using P6-C4 chemistry** Data are based on a 20 kb size-selected *E. coli* library using a 4-h movie. Each SMRT cell produces 0.5–1 billion bases. The P6-C4 chemistry is currently the most advanced sequencing chemistry offered by PacBio. The figure is adapted with permission from Pacific Biosciences [8].

An important advantage of PacBio sequencing is the read length. While the original PacBio RS system with the first generation of chemistry (C1 chemistry) generated mean read lengths around 1500 bp [7], the PacBio RS II system with the current C4 chemistry boasts average read lengths over 10 kb [5], with an N50 of more than 20 kb (that is, over half of all data are in reads longer than 20 kb) and maximum read lengths over 60 kb (Figure 4) [8]. In contrast, the maximum read length of Illumina HiSeq 2500 is only paired-end 250 bp (using Rapid Run Mode) [9]. The short read lengths of SGS are commonly unable to span repetitive regions with at least one unique flanking sequence. In these cases, the origin of a read cannot be precisely determined. The consequent multiple alignments and misalignments lead to problems in downstream analysis, including the abundance estimation and the structural variation (SV) calling. Because of the much longer read lengths of PacBio sequencing, the precise location and sequence of repetitive regions can often be resolved by unique regions within a single read. Although there exist a few extremely-large repetitive regions that are longer than PacBio reads, they could be resolvable with enough heterogeneity [10].

However, compared to SGS, the throughput of PacBio sequencing is a drawback. There are 150,000 ZMWs on a single SMRT cell, each of which can produce one subread or CCS read. Typically, only 35,000–70,000 of the 150,000 ZMW wells on a SMRT cell produce successful reads [11], due to either the failure to anchor a polymerase or the loading of more than one DNA molecule in a ZMW. Typical throughput of the PacBio RS II system is 0.5–1 billion bases per SMRT cell [8]. The entire workflow—from template preparation to primary base call analysis—takes less than one day. Although the PacBio RS II generates nearly 10 times the sequence data as the old PacBio RS system with the C1 chemistry [7], it does not yet provide the high throughput offered by SGS techniques, such as Illumina HiSeq 2500. Using the new HiSeq SBS v4 reagent kits, Illumina HiSeq 2500 produces up to 8 billion paired-end 125 bp reads (1 trillion bases) per two flow cells over a 6-day run, resulting in a daily throughput of ∼167 billion bases (using High Output Run Mode) [9].

Another notable weakness of PacBio sequencing is that the error rate of a CLR is relatively high (around 11%–15%) [12]. Because the errors are distributed randomly in CLRs, the error rate can be reduced by generating CCS reads with sufficient sequencing passes. A coverage of 15 passes yields >99% accuracy [4]. However, the number of sequencing passes and the CCS read length are a trade-off, given that the total length of a CLR is limited by the life time of the polymerase [13]. That is, longer sequences yield fewer passes in a CLR, and thus lower accuracy, and *vice versa*.

Strengths and weaknesses of SGS and PacBio sequencing are complementary, which motivated an innovative strategy, hybrid sequencing, to integrate both techniques. These approaches often involve using the high-throughput and high-accuracy short read data to correct errors in the long reads, in order to reduce the required amount of more costly long-read sequence data and to salvage the relatively long, but more error-prone, subreads. Furthermore, PacBio long reads can provide reliable alignments, scaffolds, and rough detections of genomic variants, while short reads refine the alignments/assemblies/detections to single-nucleotide resolution. The high coverage of SGS data can also be utilized in downstream quantitative analysis.

Overall, PacBio sequencing provides very long reads with a high error rate and low throughput. Its relative performance compared to first, second, and third generation sequencing platforms is shown in Table 1 [5], [9], [12], [14], [15], [16], [17], [18], [19], [20], [21], [22], [23], [24], [25], [26], [27]. PacBio RS II, using the sixth generation of polymerase and the fourth generation of chemistry (P6-C4 chemistry), provides longer average read length than SGS platforms, but it has a higher single-pass error rate and lower yield. Moreover, PacBio sequencing is also faster but more costly than most other methods.

## Applications to genome research

### *De novo* assembly

*De novo* genome assembly is one of the main applications of PacBio sequencing because long reads can provide large scaffolds. PacBio long reads overcome many limitations of genome assembly using SGS data, such as the presence of highly-repetitive genomic regions. Though the error rate of PacBio data is higher than that of SGS, increased coverage or hybrid sequencing could greatly improve the accuracy. The attempts of *de novo* genome assembly using PacBio data started from small targets, such as microbial genomes. The hierarchical genome-assembly process (HGAP) developed by Chen et al. generates *de novo* assemblies using PacBio sequencing data from a single, long-insert shotgun DNA library [28]. In addition, a few methods using hybrid sequencing data, including ALLPATHS-LG, PacBio Corrected Reads pipeline, SPAdes, and SSPACE-LongRead, have been applied to complete bacterial genome assemblies [29]. Table 2 provides a list of selected *de novo* assemblies produced using PacBio sequencing alone or using hybrid sequencing, along with some of their noteworthy achievements [7], [30], [31], [32], [33], [34], [35].

The benefits of PacBio sequencing are apparent in the *de novo* assembly produced by Brown et al., who compared PacBio sequencing to Illumina, 454, and Illumina/454 hybrid assemblies in finishing the 100-contig draft genome of *Clostridium autoethanogenum* DSM 10061 [7]. *C. autoethanogenum* is an acetogen that is capable of producing ethanol from carbon monoxide, and it is therefore of great biotechnological interest. Its genome has a 31.1% GC content and contains repeats, prophage, and nine copies of the rRNA gene operons, so it is classified as a Class III genome, the most complex genome classification in terms of repeat content and repeat type [7]. Using only one PacBio library preparation and two SMRT cells, the entire genome could be assembled *de novo* in a single contig, despite the complexity of the *C. autoethanogenum* genome. No method based on SGS data could assemble the genome in less than 22 contigs, and each of the SGS assemblies contained at least four collapsed repeat regions, while the PacBio assembly had none [7].

In addition, a *de novo* assembly of the *Potentilla micrantha* chloroplast genome using PacBio sequencing covered the entire 154,959 bp of the chloroplast genome in a single contig [33]. This offered an improvement over the Illumina assembly, which covered 90.59% of the genome in seven contigs. The PacBio assembly also revealed no bias in coverage in GC-rich regions and resolved 187 ambiguities in the Illumina assembly, including long inverted repeat regions that are characteristic of chloroplast genomes.

In contrast to relatively small genomes, the assembly of large genomes by overlapping sequence reads can be more computationally expensive. In order to overcome this obstacle, Berlin et al. developed the MinHash Alignment Process (MHAP) for efficient overlapping of noisy long reads [31]. MHAP creates a compact representation of sequencing reads by utilizing a dimensionality reduction technique called MinHash [36]. Compared to BLASR, another aligner that is capable of overlapping PacBio reads [37], MHAP efficiently constructed comparable or improved *de novo* assemblies of the human genome and the genomes of four model organisms (*Escherichia coli*, *Saccharomyces cerevisiae*, *Arabidopsis thaliana*, and *Drosophila melanogaster*) using PacBio sequencing without SGS short reads. In particular, this method resulted in a 600-times faster assembly for *D. melanogaster*. This assembly contained only 132 contigs, and it potentially resolved 52 of the 124 gaps in the version 5 reference genome of *D. melanogaster*.

The haploid human genome assembly by MHAP is highly contiguous and potentially closes 51 of 819 gaps in GRCh38 [31]. As an example of a difficult region to assemble, the major histocompatibility complex (MHC), which has an important role in immunity, was broken into over 60 contigs in the Illumina assembly, while 97% of the region was assembled in just two contigs using PacBio sequencing. PacBio long reads also allowed for the reconstruction of repetitive heterochromatic sequences in telomeric regions. In humans, the loss of telomeres has been associated with diseases, including premature aging syndromes and cancer [38]. PacBio sequencing offers an improvement over current reference genomes, in which telomeric regions are poorly annotated, which will improve the study of telomere-associated diseases.

In 2015, another *de novo* assembly of a haploid human genome by Chaisson et al. closed 50 of the 164 gaps in GRCh37 and shortened 40 other gaps [32]. 39 of the 50 closed gaps included short tandem repeats (STRs) in GC-rich regions. STRs are repetitive elements of 2–6 nucleotides that are generally not sequenceable beyond 100 bp by SGS. This assembly also identified 47,238 breakpoint positions, resolving 26,079 euchromatic structural variations (SVs) at the single-nucleotide resolution, including inversions, complex insertions, and repetitive regions.

As alternatives to using PacBio sequencing alone for eukaryotic *de novo* assemblies, error correction strategies using hybrid sequencing have also been developed. Koren et al. developed the PacBio corrected Reads (PBcR) approach for using short reads to correct the errors in long reads [34]. PBcR has been applied to the previously-unsequenced parrot (*Melopsittacus undulatus*) genome using 5.5× coverage of PacBio reads that were corrected by 15.4× coverage of 454 reads, yielding 3.83× coverage of corrected reads. The error correction required 6.8 days to complete. The >1 Gb assembly consisted of 15,328 contigs, with an N50 of 93,069 bp. Also, Bashir et al. used hybrid sequencing data to assemble the genome of a recent Haitian cholera outbreak strain at >99.9% accuracy in two nearly finished contigs, completely resolving complex regions with clinically relevant structures [30].

Using the direct sequencing protocol without constructing a library, PacBio data can be generated from as little as 1 ng of DNA, while typical protocols require 400–500 ng of sheared DNA for library preparation [39]. At the cost of reducing the yield per SMRT cell to around 3000 reads, which limits its utility to small genomes, this method allows PacBio data to be generated within eight hours of receiving the sample, less than half the time required when library preparation is included. This approach has been applied to sequence antibiotic resistance gene-carrying bacterial plasmids, plasmid vector models for analysis of DNA-modification, linear DNA fragments covering an entire bacterial genome, and single- or double-stranded viral genomes [39]. Because it requires no *a priori* knowledge of any sequence or organism-specific reagents but offers the high speed and low DNA requirement of direct sequencing, this method could be applicable to sequence plasmids, viruses, mitochondrial DNA, and microbial pathogens in a clinical setting.

### Problematic genomic regions

Closing gaps in draft genomes can also be accomplished efficiently via PacBio sequencing of PCR products. This approach is more cost-effective than Sanger sequencing and is able to close gaps greater than 2.5 kb in a single round of reactions [40]. However, a loading bias against larger PCR products is present since smaller PCR products load into the ZMWs with greater efficiency. This bias can be reduced if the molar ratio of the PCR products is adjusted according to their size and concentration when pooling them together. Zhang et al. compared this gap closure method with Sanger sequencing for 362 gaps ranging from 500 bp to 5 kb from 16 diverse genomes [40]. Of gaps smaller than 2.5 kb, 64% and 73% were closed by Sanger and PacBio sequencing, respectively, while none of the gaps larger than 2.5 kb were closed by Sanger sequencing, compared to 88% by PacBio sequencing. They also found that only the PacBio platform could sequence through small hairpin structures (called hard stops) and that the PacBio platform performed better in high GC regions compared to Sanger sequencing.

STRs are associated with many genetic disorders and are difficult to detect with SGS technologies. One such gene is the human fragile X mental retardation 1 (*FMR1*) gene. *FMR1* contains a (CGG)_n_ repeat that is responsible for heritable disorders including fragile X syndrome, fragile X-associated tremor/ataxia syndrome, adult-onset neurodegenerative disorder, premature ovarian insufficiency, learning disabilities, autism spectrum disorders, attention deficit hyperactivity disorder, and seizures [41]. There are normally 7–60 (CGG) repeats, while the permutation range is 60–230 repeats, and the full mutation range is over 230 repeats [42]. Loomis et al. generated PacBio long reads for expanded CGG-repeat *FMR1* alleles in full mutation range [41]. They demonstrated that PacBio sequencing was not adversely affected by expansions exceeding 750 repeats, suggesting that productive sequencing is limited only by factors governing the productive lifetime of the polymerase and the desired number of subreads within an individual CCS read. PacBio targeted sequencing has also been used to resolve the genomic gap in *MUC5AC* [43], which encodes a large, secreted mucin that is important in cystic fibrosis, lung cancer, and respiratory diseases [44]. By sequencing PCR products covering the central exon, SVs among four individuals were also characterized [43].

While the examination of STRs in *FMR1* and *MUC5AC* made use of PacBio sequencing alone, Doi et al. developed a method for rapidly finding long STRs in personal genomes using hybrid sequencing [45]. They applied this method to locate an STR associated with the brain disease, spinocerebellar ataxia 31 (SCA31). Using PacBio sequencing targeting this site, they revealed that the instability of the repeat expansions associated with SCA31 is determined by (TGGAA) and (TAAAATAGAA) repeats.

These successful applications indicate the promising utility of PacBio sequencing for the study of other diseases, such as myotonic dystrophy, Huntington’s disease, Friedreich’s ataxia, and amyotrophic lateral sclerosis-frontal temporal dementia (ALS-FTLD), which are all associated with repeat expansions [41]. In contrast to SGS, PacBio sequencing is able to obtain information from individuals with expanded STRs and could likely be developed as a diagnostic approach.

### Characterization of structural variation

Compared to SNPs, large structural variations (SVs), such as copy-number variations (CNVs), copy-number neutral inversions, mobile-element insertions (MEIs), deletions, translocations, and combinations of these events, are more challenging to detect and characterize. Characterization of SVs is crucial to the study of many diseases, including cancer [46], [47]. Up to 13% of the human genome is subject to SVs [48], which account for a majority of variant bases. In the Sanger-sequenced diploid genome of an individual human, 74% of a total of 12.3 Mb of variant bases were SVs [49]. However, due to the short sequencing length, the SGS approaches impose severe limitations on the study of these complex SVs, particularly those involving repetitive regions.

PacBio sequencing is based on single-molecule sequencing technology and provides much longer reads. Thus, it is adept at identifying non-SNP DNA variations, albeit at the cost of higher per-nucleotide error rates. In 2014, a SV detection tool, MultiBreak-SV, was developed to analyze PacBio sequencing data, paired-end short reads, or hybrid sequencing data [50]. Ritz et al. demonstrated that MultiBreak-SV is able to detect SVs with high sensitivity and specificity by applying to PacBio data from four human fosmids. They also predicted 1002 SVs in a hydatidiform mole genome (CHM1tert) using PacBio data, over half of which were confirmed by an Illumina assembly [50].

Cancer development has been attributed to SVs including large chromosomal rearrangements, duplications, and deletions. While recurring SVs may be viable biomarkers for disease detection and prognosis, they are difficult to monitor when the breakpoint of the SV is unknown. In many cases, such as the *CDKN2A* deletion, which diminishes expression of multiple tumor-suppressor proteins, the breakpoints may vary between individuals [46]. Patel et al. developed Amplification of Breakpoints (AmBre), a pipeline to identify DNA breakpoints associated with known translocations and deletions using PacBio sequencing [46]. Using AmBre, they discovered *CDKN2A* deletion breakpoints in six cancer cell lines, including MCF7, for which previous studies have failed to annotate the *CDKN2A* breakpoints, likely due to repetitive sequences [46], [51], [52].

The first long-read characterization of SV in a personal diploid human genome has been recently performed with Parliament, a consensus SV-calling infrastructure that utilizes multiple SV detection methods and data types, including PacBio long reads [48]. Parliament identified over 31,007 genomic loci ranging between 100 bp and 1 Mb from a single individual (HS1011) that deviated from the hg19 reference assembly. Among them, 9777 loci, which span 59 Mb of the reference genome (1.8%), were corroborated as SVs by PacBio sequencing, local hybrid sequencing, or multi-source heuristics. Of these 9777 loci, 3801 loci were identified only by long-read data.

## Applications to transcriptome research

### Transcript sequencing using Iso-Seq

Understanding the complete expression of gene isoforms (*i.e.*, transcripts) is fundamental for transcriptome studies. While SGS is frequently used for gene profiling, it is often unable to identify full-length gene isoforms and can introduce amplification bias. SGS faces particularly severe limitations on transcript recall and splice product discrimination in the context of complex eukaryotic genomes. An assessment of SGS methods for transcript reconstruction found that expression level estimates varied widely across methods, even when based on similar transcript models [53]. Because PacBio sequencing produces longer reads, it can be used to more comprehensively identify transcripts.

Pacific Biosciences developed a protocol, Iso-Seq, for transcript sequencing, including library construction, size selection, sequencing data collection, and data processing. Iso-Seq allows direct sequencing of transcripts up to 10 kb without use of a reference genome [54]. The experimental component of Iso-Seq is to select and sequence full-length transcripts, and the following data processing step generates the highest-quality reads of each selected transcript, called “Reads of Insert.” Iso-Seq has been used to characterize alternative splicing events involved in the formation of blood cellular components [55]. This is critical for interpreting the effects of mutations leading to inherited disorders and blood cancers, and can be applied to design strategies to advance transplantation and regenerative medicine. In addition, using PacBio sequencing on the polyadenylated RNA complement of 20 human organs and tissues, Sharon et al. obtained 476,000 CCS reads and identified ∼14,000 spliced GENCODE genes. Interestingly, over 10% of their alignments represent previously-unannotated intron structures [56].

Nonetheless, the sensitivity of Iso-Seq method is limited by the following factors: (1) the selection of full-length transcripts is not complete, so not all Reads of Insert represent full-length transcripts; (2) very long transcripts are likely not sequenced in full due to the sequencing length limit; (3) high-quality reads (CCS reads) can be generated only if the target cDNA is short enough to be sequenced by multiple passes. As Pacific Biosciences has been improving the throughput and sequencing movie time, the limitation could be reduced, though not thoroughly. An alternative way to overcome this limitation is to integrate SGS short reads and PacBio long reads via hybrid sequencing.

### Gene isoform identification using hybrid sequencing

Besides genome assembly, hybrid sequencing can also be applied to the error correction of PacBio long reads of transcripts. Moreover, it could improve gene isoform identification and abundance estimation. Au et al. have developed the tool LSC for the correction of raw PacBio reads by SGS short reads [57]. Applying this tool to 100,000 human brain cerebellum PacBio subreads and 64 million 75-bp Illumina short reads, they reduced the error rate of the long reads by more than 3-fold. In order to identify and quantify full-length gene isoforms, they also developed an Isoform Detection and Prediction tool (IDP), which makes use of TGS long reads and SGS short reads [58]. Applying LSC and IDP to PacBio long reads and Illumina short reads of the human embryonic stem cell (hESC) transcriptome, they detected 8084 RefSeq-annotated gene isoforms at full-length and predicted an additional 5459 gene isoforms through statistical inference [58]. Compared to Cufflinks, a widely-used tool for gene isoform identification and quantification based on SGS short reads [59], IDP had a much higher sensitivity for isoform identification (62% true positive for IDP *vs.* 20% true positive for Cufflinks) at a 5% false positive rate. Over one-third of the 5459 isoforms predicted by IDP were novel, and 273 of these were transcribed from 216 unannotated gene loci. The improved identification of the hESC transcriptome obtained through hybrid sequencing will facilitate the development of models of differentiation and cell commitment within the developing embryo and a better understanding of the molecular mechanisms involved in the maintenance of pluripotency. IDP-fusion has also been recently released for the identification of fusion genes, fusion sites, and fusion gene isoforms from cancer transcriptomes [60]. In the human MCF7 breast cancer cell line, IDP-fusion detected fusion genes at a much higher precision than non-hybrid tools that use TGS alone and SGS alone (69% true positive for IDP-fusion *vs.* 31% for TRUP [61], 23% for TopHat-Fusion [62], and 21% for Iso-Seq [54]), with similar sensitivity.

### Personal transcriptomes

Personal transcriptomes are expected to have applications in understanding individual biology and disease, but SGS has been shown to be insufficiently accurate for the identification and quantification of an individual’s genetic variants and gene isoforms [53], [63], [64]. Using a hybrid sequencing strategy combining PacBio long reads and Illumina short reads, Tilgner et al. sequenced the lymphoblastoid transcriptomes of three family members in order to produce and quantify an enhanced personalized genome annotation [63]. Around 711,000 CCS reads were used to identify novel isoforms, and ∼100 million Illumina paired-end reads were used to quantify the personalized annotation, which cannot be accomplished by the relatively small amount of long reads alone. This method produced reads representing all splice sites of a transcript for most sufficiently expressed genes shorter than 3 kb. It provided a *de novo* approach for determining single-nucleotide variations (SNVs), which could be used to improve RNA haplotype inference. By additionally producing transcriptomes for both parents, they found that PacBio sequencing improved the accuracy of personal transcriptomes despite the high error rate of PacBio data. Single molecules can be attributed to the allele from which they were transcribed, which could also allow for the assessment of biased allelic or isoform expression.

## Applications to epigenetics research

DNA modifications can influence a variety of processes in many organisms, including gene expression, gene silencing, host–pathogen interactions, and DNA replication, repair, and transcription regulation [65]. In bacterial genomes, *N*^6^-methyladenine (m^6^A), *N*^4^-methylcytosine (m^4^C), and 5-methylcytosine (m^5^C) function as components of restriction-modification (RM) systems [66], [67], [68], [69]. Along with m^6^A and m^5^C, modified bases such as 5-hydroxymethylcytosine (5-hmC), 5-formylcytosine (5fC), and 5-carboxylcytosine (5caC) are also present in eukaryotic genomes [68]. However, because SGS lacks simple methods to determine the locations of most DNA modifications, many DNA modifications have typically been ignored.

Cytosine methylation is the most widely-studied DNA modification. Bisulfite sequencing is the most common high-throughput sequencing method for genome-wide detection of these epigenetic events, typically using Illumina short reads [70]. This method involves treating DNA with a bisulfite reagent that converts unmethylated cytosine to uracil, but it requires a well-defined reference genome [70]. The sample preparation steps can be costly and time-consuming, and the required reaction conditions can degrade DNA [71]. Bisulfite sequencing is also limited to the detection of specific forms of methylation that can undergo this conversion [70]. More importantly, it cannot discriminate between C, m^5^C, and 5hmC [67].

**Figure 5:**
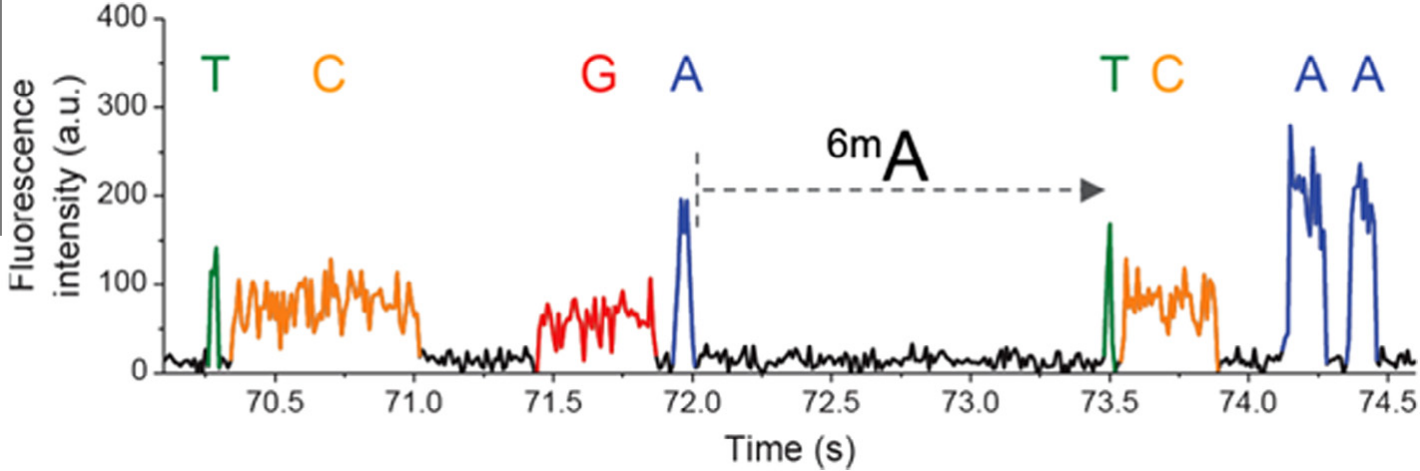
**Detection of methylated bases using PacBio sequencing** PacBio sequencing can detect modified bases, including m^6^A (also known as ^6m^A), by analyzing variation in the time between base incorporations in the read strand. The figure is adapted with permission from Pacific Biosciences [72]. a.u. stands for arbitrary unit.

In contrast to base conversion employed in bisulfite sequencing, PacBio sequencing uses an alternative approach to directly detect native epigenetic modifications. It monitors time between base incorporations in the read strand, called inter-pulse durations (IPDs). The difference of IPDs between normal and modified bases serves as signal to detect base modifications (Figure 5) [72]. This technique is applicable to the detection of either DNA or RNA modifications [73] and overcomes many of the limitations and burdens of bisulfite sequencing, with unique kinetic characteristics observable for over 25 types of base modifications [67]. However, because the kinetic variation signals produced by m^5^C modifications are very weak, PacBio sequencing is unable to detect m^5^C modifications with high accuracy [66], [68].

The resequencing of six bacteria, including *Geobacter metallireducens* GS-15, *Chromohalobacter salexigens*, *Vibrio breoganii* 1C-10, *Bacillus cereus* ATCC 10987, *Campylobacter jejuni* subsp. jejuni 81-176, and *C. jejuni* NCTC 11168, by Murray et al. using PacBio sequencing resulted in the discovery of new m^6^A and m^4^C methylation patterns in each genome [68]. The m^6^A and m^4^C methyltransferases (MTases) responsible for those patterns were also assigned. This study showed that PacBio sequencing provides information about not only which MTase genes are active, but also their recognition sequences by aligning the methylated bases with their kinetic signatures.

Using kinetic variation data obtained via PacBio sequencing, Fang et al. detected 49,311 m^6^A residues and 1407 m^5^C residues in a pathogenic *E. coli* genome [66]. They were also able to deduce the target sites of MTases that catalyze m^6^A modifications using PacBio sequencing kinetic variation data alone. In addition, they found that an MTase component of an RM system affected gene expression and DNA replication, suggesting that the RM systems function beyond protecting host genomes from foreign DNA.

The detection of epigenetic motifs using PacBio sequencing is not limited to settings with complete references or high-coverage samples. Beckmann et al. have demonstrated the ability of PacBio sequencing to recover previously-discovered epigenetic motifs with m^6^A and m^4^C modifications in both low-coverage and high-contamination scenarios [70]. They were also able to recover many motifs from three mixed strains (*E. coli*, *G. metallireducens*, and *C. salexigens*), even when the motif sequences of the genomes of interest overlap substantially, suggesting that PacBio sequencing is applicable to metagenomics. They note that hybrid sequencing would be more cost-effective than using PacBio sequencing alone in order to detect and accurately define k-mers for low proportion genomes.

Epigenetic motifs can also be detected in conjunction with *de novo* assembly using PacBio sequencing, as was done for *Helicobacter pylori*. *H. pylori* is a bacteria found in stomachs of about two-thirds of the world’s population that can cause ulcers and lead to stomach cancer [74]. Its genome is around 1.6 Mb in size with a GC content of 39% and high allelic diversity. Whole-genome sequencing by PacBio platform of eight *H. pylori* strains has recently determined a single, complete contig for each strain through *de novo* assembly [35]. Moreover, in this study, the methylation information provided by PacBio sequencing additionally led to the identification of epigenetic motifs that were associated with virulence factors.

In order to increase the detection accuracy of DNA modifications, and to reduce or eliminate the required coverage of control data that are free of modifications, Feng et al. developed an empirical Bayesian hierarchical model for incorporation of historical PacBio sequencing data [75]. Because local sequence context can explain roughly 80% of the variation in polymerase kinetics near a given incorporation site [75], they estimate an expected kinetic rate of the polymerase at that incorporation site using historical data. They demonstrated that this method can increase detection accuracy at a reduced sequencing cost by applying their model to detect modifications in plasmids with known modified sites and an *E. coli* K-12 strain. This model has been implemented in the R package “seqPatch” (available at https://github.com/zhixingfeng/seqPatch).

Intercellular heterogeneity, *i.e.*, differential DNA modification status between cells in a population, is a major cause of phenotypic heterogeneity in many organisms [76]. In order to quantitatively detect intercellular heterogeneity in genome DNA modifications, Feng et al. developed qDNAmod, a bioinformatics tool for analysis of PacBio sequencing data [76]. Applying qDNAmod to *Streptococcus pneumoniae* strain ST556, Feng et al. determined that four highly-significant methylation motifs contained m^6^A. They mapped these motifs to the genome and found that the intercellular heterogeneity of the methylation is mediated by two type I RM systems. Therefore, their studies demonstrate that investigation of intercellular heterogeneity in previously undetectable genome DNA modifications (such as m^6^A and m^4^C) is facilitated by the direct detection of modifications in single molecules by PacBio sequencing.

## Discussion

In the past years, PacBio sequencing has been applied to *de novo* assemble or resequence a variety of genomes (from small microbe to human), characterize the complexity of transcriptomes at the isoform level, and study base modifications. A summary of the advantages and achievements of PacBio sequencing in recent years is given in Table 3 [7], [30], [31], [32], [33], [34], [35], [40], [41], [43], [45], [46], [48], [50], [55], [56], [58], [60], [63], [66], [67], [68], [70], [75], [76].

In the meantime, a number of relevant bioinformatics tools have also been developed. As demonstrated by these recent studies, PacBio sequencing provides an unprecedented opportunity to overcome many of the obstacles faced by SGS via providing longer read lengths, kinetic variation information, and shorter run times, yet the technology still has room for improvement in other aspects, such as the high error rate of raw single-pass data. Obtaining sufficient read depth to build a sufficiently-accurate consensus sequence can be costly for large, complex genomes. On the other hand, obtaining high coverage for smaller genomes is more affordable. For microbial genomes, 100× coverage is reliable for resolving repetitive regions and costs less than $1000 with a 20 kb library preparation [6]. Although PacBio is still outperformed by SGS with regard to throughput, the sequencing chemistry, protocol workflows, and software continue to improve. One such improvement will be active loading to increase the rate of successfully loading a single polymerase in each ZMW well [77]. In the near future, neither PacBio sequencing nor SGS is likely to be replaced by the other.

As PacBio sequencing and SGS each have their own strength and weakness, hybrid sequencing has become a more popular approach to fully make use of the advantages of both platforms. In particular, hybrid sequencing makes the cost and work load more acceptable for small-size biomedical research laboratories compared to PacBio sequencing alone, and it brings exclusive information that is not available from SGS. The long-read PacBio data shed light on intractable problems in SGS, while the research outcome can be quantified or refined to single-nucleotide resolution via integration with high-throughput and high-accuracy SGS data.

While PacBio sequencing may coexist with and complement SGS, it faces the competition from the other developing TGS technologies, such as Oxford Nanopore Technologies (ONT). ONT is the second commercialized TGS platform from 2014. Although only very limited studies and data of ONT have been published, it shows similar advantages and disadvantages: ONT reads are long but are of even higher error rate than PacBio sequencing [22]. However, unlike PacBio’s CCS strategy of repeatedly sequencing a target with many passes to improve accuracy, ONT is limited to only two passes by design, so its high error rate is a major disadvantage. While the read lengths in many of the current studies using ONT have not surpassed those of PacBio sequencing, ONT could potentially offer longer average reads than PacBio. On top of that, ONT MinION™ is a portable and very small USB device, which makes it stand out from all existing sequencing technologies. Moreover, the far lower price ($1000 in ONT early access program) [78] could make ONT as a routine experimental measure in biomedical laboratories.

In January of 2015, Dr. Jonas Korlach, Chief Scientific Officer of Pacific Biosciences, predicted that the throughput of PacBio sequencing will increase 4-fold in 2015, reaching at least 4 Gb per SMRT cell run, and that the average read lengths of PacBio sequencing will reach 15–20 kb during 2015 [77]. In September, the company announced its new Sequel System, which will feature one million ZMWs per SMRT cell instead of 150,000 and therefore deliver seven times more reads per SMRT cell, according to PacBio [79], [80]. It will also offer the flexibility of up to 16 SMRT cells per run [80]. The Sequel System will cost $350,000, less than half the cost of the PacBio RS II ($750,000), and PacBio CEO Mike Hunkapiller claims that the system will be able to deliver a 10× human genome in one day at a consumables cost of $3000 [79]. According to Dan Zabrowski of Roche, which partnered with Pacific Biosciences to develop the Sequel System, this system will serve as the basis for a series of clinical platforms in late 2016, paving the way for PacBio sequencing to be used for diagnostics [79]. Although PacBio sequencing has shown advantages in some unique niches, the cost and throughput have previously prevented it from being applied more broadly since many biomedical researches focus on the large and complicated human genome. The Sequel System’s increased throughput and decreased cost have the potential to remedy this limitation.

## Competing interests

The authors have declared no competing interests.

## Figures and Tables

**Table 1:** Performance comparison of sequencing platforms of various generations

**Method**	**Generation**	**Read length (bp)**	**Single pass error rate (%)**	**No. of reads per run**	**Time per run**	**Cost per million bases (USD)**	**Refs.**
Sanger ABI 3730×l	1st	600–1000	0.001	96	0.5–3 h	500	[14], [18], [19], [20], [21]
Ion Torrent	2nd	200	1	8.2 × 10^7^	2–4 h	0.1	[15], [25]
454 (Roche) GS FLX+	2nd	700	1	1 × 10^6^	23 h	8.57	[14], [17], [27]
Illumina HiSeq 2500 (High Output)	2nd	2 × 125	0.1	8 × 10^9^ (paired)	7–60 h	0.03	[9], [16], [26]
Illumina HiSeq 2500 (Rapid Run)	2nd	2 × 250	0.1	1.2 × 10^9^ (paired)	1–6 days	0.04	[9], [16], [26]
SOLiD 5500×l	2nd	2 × 60	5	8 × 10^8^	6 days	0.11	[14], [24]
PacBio RS II: P6-C4	3rd	1.0–1.5 × 10^4^ on average	13	3.5–7.5 × 10^4^	0.5–4 h	0.40–0.80	[5], [12], [15]
Oxford Nanopore MinION	3rd	2–5 × 10^3^ on average	38	1.1–4.7 × 10^4^	50 h	6.44–17.90	[22], [23]

**Table 2:** *De novo* genome assemblies using hybrid sequencing or PacBio sequencing alone

**Species**	**Method**	**Tools**	**SMRT cells**	**Coverage**	**Contigs**	**Achievements**	**Ref.**
*Clostridium autoethanogenum*	PacBio	HGAP	2	179×	1	21 fewer contigs than using SGS; no collapsed repeat regions (⩾4 using SGS)	[7]
*Potentilla micrantha* (choloroplast)	PacBio	HGAP, Celera, minimus2, SeqMan	26	320×	1	6 fewer contigs than with Illumina; 100% coverage (Illumina: 90.59%); resolved 187 ambiguous nucleotides in Illumina assembly; unambiguously assigned small differences in two >25 kb inverted repeats	[33]
*Escherichia coli*	PacBio	PBcR, MHAP, Celera, Quiver	1	85×	1	4.6 CPU hours for genome assembly (10× improvement over BLASR)	[31]
*Saccharomyces cerevisiae*	PacBio	PBcR, MHAP, Celera	12	117×	21	27 CPU hours for genome assembly (8× improvement over BLASR); improved current reference of telomeres	[31]
*Arabidopsis thaliana*	PacBio	PBcR, MHAP, Celera	46	144×	38	1896 CPU hours for genome assembly	[31]
*Drosophila melanogaster*	PacBio	PBcR, MHAP, Celera, Quiver	42	121×	132	1060 CPU hours for genome assembly (593× improvement over BLASR); improved current reference of telomeres	[31]
*Homo sapiens* (CHM1htert)	PacBio	PBcR, MHAP, Celera	275	54×	3434	262,240 CPU hours for genome assembly; potentially closed 51 gaps in GRCh38; assembled MHC in 2 contigs (60 contigs with Illumina); reconstructed repetitive heterochromatic sequences in telomeres	[31]
*Homo sapiens* (CHM1tert)	PacBio	BLASR, Celera, Quiver	243	41×	N/A (local assembly)	Closed 50 gaps and extended into 40 additional gaps in GRCh37; added over 1 Mb of novel sequence to the genome; identified 26,079 indels at least 50 bp in length; cataloged 47,238 SV breakpoints	[32]
*Melopsittacus undulatus*	Hybrid	PBcR, Celera	3	5.5× PacBio + 15.4× 454 = 3.83× corrected	15,328	1st assembly of >1 Gb parrot genome; N50 = 93,069	[34]
*Vibrio cholerae*	Hybrid	BLASR, Bambus, AHA	195	200× PacBio + 28× Illumina + 22× 454	2	No N’s in contigs; 99.99% consensus accuracy; N50 = 3.01 Mb	[30]
*Helicobacter pylori*	PacBio	HGAP, Quiver, PGAP	8 per strain	446.5× average among strains	1 per strain	1 complete contig for each of 8 strains; methylation analysis associated motifs with genotypes of virulence factors	[35]

*Note:* N50, the contig length for which half of all bases are in contigs of this length or greater; MHC, major histocompatibility complex; SV, structural variation.

**Table 3:** Summary of PacBio sequencing applications and main achievements

**Application**	**Genome research**	**Transcriptome research**	**Epigenetics research**
Advantage	Closes gaps and completes genomes due to longer reads	Identifies full-length transcript isoforms without need for a reference genome	Detects modifications by monitoring kinetic variation
	Identifies non-SNP SVs	Detects novel isoforms and fusion events	Detects epigenetic motifs in low coverage settings and with mixed genomes
Achievements	Produced highly-contiguous assemblies of bacterial and eukaryotic genomes	Identified previously-unannotated human intron structures	Discovered new m^6^A and m^4^C MTases and methylation patterns in 6 bacteria
	Discovered STRs and mutations associated with *FMR1*, brain disease, cystic fibrosis, lung cancer, and respiratory diseases	Characterized alternative splicing events involved in the formation of blood cellular components	Detected m^6^A and m^5^C residues in *Escherichia coli*; deduced target sites of MTases that catalyze m^6^A modifications
	Discovered *CDKN2A* deletion breakpoints in six cancer cell lines	Identified novel isoforms in hESC transcriptome using hybrid sequencing	Identified virulence factor genotype-dependent motifs in *Helicobacter pylori*
	Characterized SVs in a personal diploid human genome	Quantified personal transcriptome, including novel isoforms, splice sites, and SNVs	Detected intercellular heterogeneity in genome DNA modifications in *Streptococcus pneumoniae*
Refs.	[7], [30], [31], [32], [33], [34], [40], [41], [43], [45], [46], [48], [50]	[55], [56], [58], [60], [63]	[35], [66], [67], [68], [70], [75], [76]

*Note:* STR, short tandem repeat; FMR1, fragile X mental retardation 1; CDKN2A, cyclin-dependent kinase inhibitor 2A; SV, structural variation; SNV, single nucleotide variation; MTase, methyltransferase; hESC, human embryonic stem cell.
